# Multi-Objective Optimization and Performance Evaluation of Rhombic Pin-Fin Microchannel Heat Sinks with Diverse Manifold Configurations

**DOI:** 10.3390/mi17020273

**Published:** 2026-02-23

**Authors:** Ruicheng Rong, Xiangqi Liu, Xiao Jin, Ruijin Wang

**Affiliations:** 1 School of Mechanical Engineering, Hangzhou Dianzi University, Hangzhou 310018, China; 2 Information Engineering College, Hangzhou Dianzi University, Hangzhou 310018, China

**Keywords:** microchannel heat sink, rhombic pin-fin, thermal performance, trapezoidal manifold, multi-objective optimization

## Abstract

In response to the increasingly severe heat dissipation challenges in electronic devices, three types of manifold microchannel heat sinks (MMC) incorporating rhombic pin-fins were proposed. Under the constraint that the maximum temperature of the heat source surface remains below 343.15 K, numerical comparisons with a conventional straight rectangular microchannel heat sinks (MCHS) reveal that the design featuring a trapezoidal manifold exhibits superior comprehensive thermal performance and improved temperature uniformity. Furthermore, the influence of rhombic pin-fin geometry on thermal performance was investigated for both MCHS with and without the trapezoidal manifold under varying mass flow rates. Results show that for the MCHS without a manifold, performance evaluation criterion (PEC) reaches its maximum when the inlet angle of the rhombic pin-fin is 120°, the side length is 0.17 mm, and the pin-fin height is 0.18 mm. In contrast, for the MCHS with the trapezoidal manifold, optimal PEC is achieved at an inlet angle of 110°, a side length of 0.18 mm, and a pin-fin height of 2.2 mm. Additionally, a multi-objective optimization was conducted using the Latin hypercube sampling method. Three objective functions—maximum temperature ($T_{\max}$), thermal performance (PEC), and temperature uniformity ($\sigma_{T}$)—were considered. A total of 150 sample points were used to train Kriging surrogate models for the rhombic pin-fin MCHS with trapezoidal manifold. The optimization results demonstrate a 34.05% enhancement in thermal performance and an 18.6% improvement in temperature uniformity.

## 1. Introduction

In recent years, the rapid advancement of artificial intelligence (AI) has accelerated the demand for high power-consumption CPUs and GPUs. For example, NVIDIA’s high-performance GPU H100 SXM features a maximum thermal design power of 700 W, accompanied by a surface heat flux density ranging from 80 to 100 W/cm^2^ [1]. Traditional heat transfer technologies struggle to accommodate levels as high as 1000 W/cm^2^ [2]. Research has demonstrated that at temperature between 70 and 80 °C, for every 1 °C increase in temperature, the reliability of the electronic device decreases by approximately 5%, with the failure rate almost increases exponentially with the temperature [3]. Currently, inadequate heat dissipation has emerged as a primary factor contributing to chip failures. The concept of MCHS was first introduced by Tuckerman in 1981. Experimental findings reveal that MCHS can effectively cool electronic devices with a maximum heat flux density of 790 W/cm^2^ [4]. Initially, early microchannel cross-sections were predominantly designed as straight rectangular structures. Subsequent research efforts have focused on enhancing the overall flow channel design within these microchannels; this includes optimizing the width-to-depth ratio of flow channels [5], altering cross-sectional shapes to include rhombic, circular, triangular, trapezoidal configurations, among others [6], and designing flow paths with stepped, sawtooth or wave-like geometries [7]. It has been observed that when fluid flows through a straight channel stably, boundary layer growth leads to a deterioration in heat transfer performance. Consequently, many researchers have incorporated smaller channels between main channels or implemented multi-layer structures to generate secondary flows aimed at enhancing fluid convection or periodically disrupting boundary layers to improve both thermal performance and temperature uniformity within MCHS systems [8,9]. Further research has demonstrated that the protruding structures on the inner wall of microchannels, such as porous ribs, triangular ribs, and offset ribs, can disrupt the boundary layer, induce disturbances, and enhance heat transfer [10,11]. Our previous study has identified four mechanisms that enhance the thermal conductivity of MCHS: convection enhancement, disruption of the thermal boundary, an increase in interface area, and improvement of fluid thermal conductivity [12].When incorporating pin-fin structures within the channel, common geometric configurations include rectangles, circles, triangles, squares, rhombuses, regular polygons, and trapezoids [13,14]. These pin-fin structures continuously disturb the boundary layer and generate vortices around them to improve convective heat transfer [15]. Notably, rhombic pin-fins exhibit superior heat transfer performance compared to other conventional geometric shapes under equivalent pressure drops [16]. Furthermore, specialized pin-fin designs such as Chevron [17], Oblong shapes, fish fin-like forms, and water droplet shapes [18] can further minimize flow resistance while enhancing the overall performance of MCHS. Additionally, optimizing both the shape and geometric dimensions of these pin-fins or ribs can significantly elevate thermal performance in MCHS applications.

Incorporation of a manifold structure atop the traditional MCHS (referred to as a manifold microchannel heat sink (MMC)) has proposed to shorten the flow path of the fluid, thereby reducing the overall pressure drop associated with MMCs [19]. To evaluate the enhancement in heat transfer performance offered by MMCs compared to conventional MCHSs, R. van Erp et al. [20] developed an integrated manifold microchannel cooling structure. Their findings demonstrated that such an MMC can effectively cool microchips at power densities of 1700 W/cm^2^ while requiring only 0.57 W/cm^2^ of pump power—an efficiency that is 50 times greater than that of traditional straight MCHSs. Kong et al. [21] investigated the influence of manifold geometry and inlet mass flow rate on the thermal and hydraulic performance of MMC. Compared to conventional MCHS, the optimized MMC design reduced pressure drop by 90% and enhanced the PEC by 139%. However, non-uniform flow distribution within the MMC resulted in degraded cooling performance near the manifold outlet. To address this issue, several studies have attempted to improve flow uniformity by modifying the manifold configuration. Common designs include Z-type, C-type, H-type, U-type, ZU-type, and HU-type arrangements. Mandel et al. [22] numerically simulated three of these configurations, such as Z-type, C-type, and H-type. Under single-phase conditions, they found that the Z-type manifold offers better flow distribution at low flow rates, whereas the C-type performs better at high flow rates due to a more uniform pressure gradient between the inlet and outlet. The H-type manifold demonstrated the most uniform global flow distribution under both single-phase and two-phase conditions. Lin et al. [23] conducted a comprehensive performance study of all six manifold configurations. They reported that under single-phase flow conditions, U-type, ZU-type, and HU-type MMCs—which feature a greater number of outlets—exhibited more uniform flow distribution and comparable thermal resistance compared to Z-type, C-type, and H-type MMCs with fewer outlets. However, although the U-type manifold achieved the most uniform flow distribution, it also incurred the highest pressure drop. In contrast, ZU-type and HU-type MMCs demonstrated a more balanced overall performance. To further improve flow uniformity, some researchers have proposed convergent-shaped manifold designs. For instance, Tang et al. [24] developed a conical manifold that promoted uniform flow distribution among the channels and reduced the maximum temperature of the cover plate by approximately 24 K. Similarly, Chen et al. [25] numerically compared the performance of rectangular, stepped, trapezoidal, parabolic, and elliptical manifold designs. Their results indicated that the parabolic manifold achieved the highest performance evaluation criterion (PEC), while the trapezoidal design yielded the lowest pressure drop. These findings suggest that controlling the convergence profile of the manifold is an effective strategy for achieving uniform flow distribution in MMCs. Tang [26] combined divergent/convergent manifolds with microchannels and conducted numerical simulations to evaluate their thermal performance. Under this configuration, the thermal resistance was reduced by 19.18% relative to the conventional MMC, with improved temperature uniformity observed across the heated surface. Meanwhile, Yang [27] introduced a secondary flow channel design into the MMC architecture, developing a structure that integrates manifolds with alternately inclined microchannels. Numerical results demonstrated that, compared to the classic MMC, the optimized design achieved a 1.91% reduction in pressure drop and a 19.15% reduction in thermal resistance. Additionally, Pan [28] proposed a pin-fin interleaved MMC configuration, which combines an array of cylindrical pin-fins with a manifold system. Simulation results indicated that, under identical pressure drop conditions, the pin-fin interleaved MMC exhibited superior heat dissipation performance and enhanced temperature uniformity compared to the conventional design. Furthermore, the temperature difference across the heated surface decreased as the pressure drop increased. The lowest maximum temperature on the heated surface was achieved when the length-to-width ratio of the partitioned plate within the manifold was set to 0.32.

In conclusion, the optimization of MCHS primarily focuses on modifying flow patterns through tailored channel structures. Current integrations of manifolds with complex microchannels have largely been limited to U-shaped flow designs. However, realizing U-shaped flow in practice often requires multi-layer flow distribution, which significantly increases the manufacturing complexity and cost of MMC, while also reducing experimental reliability. Previous studies have demonstrated that rhombic pin- fins exhibit exceptional heat transfer performance. Moreover, the rhombic pin-fin features a geometrically regular shape, making it readily manufacturable using conventional microfabrication techniques, including micro-milling, micro-electrical discharge machining, and deep reactive ion etching. To leverage these advantages, a novel manifold rhombic pin-fin microchannel heat sink is proposed in this work. Parametric studies and multi-objective optimization were conducted to enhance thermal performance and minimize pumping power.

## 2. Geometrical Model

**Figure 1:**
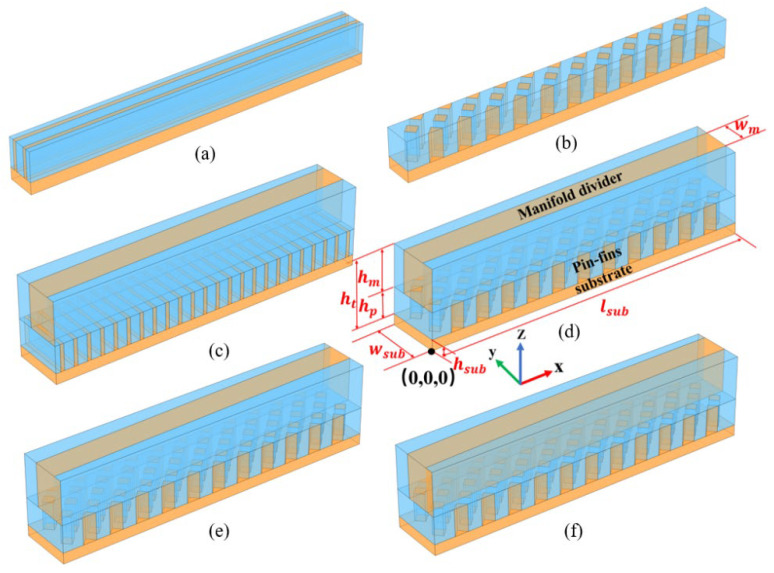
Six models for different micro heat sinks, blue and orange represent liquid and solid, respectively: (**a**) SRS, (**b**) RPS, (**c**) TMSRS, (**d**) RMRPS, (**e**) TMRPS, (**f**) CMRPS.

**Figure 2:**
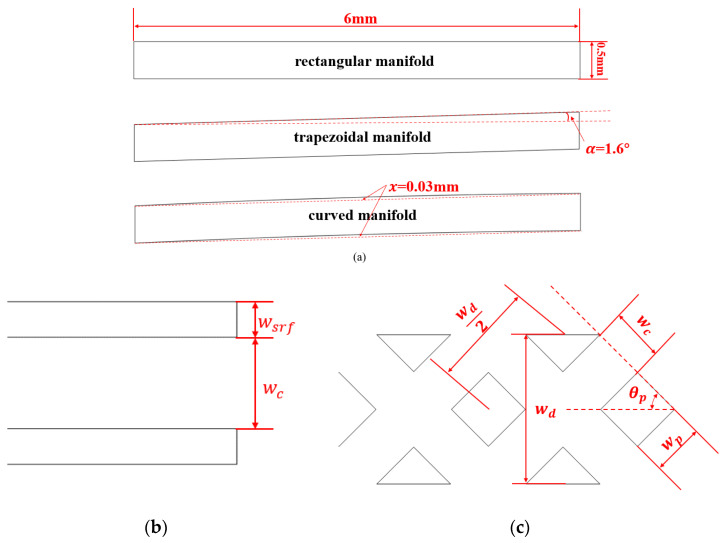
The geometric dimensions of different manifold structures (**a**), microchannel, the dot lines indicate the deviation from the rectangular one. (**b**) and rhombic pin-fin (**c**).

To visually evaluate the thermal performance of the manifold rhombic pin-fin microchannel heat sink (MRPS), six distinct micro heat sink models were constructed: a rhombic pin-fin sink (RPS), a straight rectangular sink (SRS), a trapezoidal manifold straight rectangular sink (TMSRS), a trapezoidal manifold rhombic pin-fin sink (TMRPS), a rectangular manifold rhombic pin-fin sink (RMRPS), and a curved rectangular manifold rhombic pin-fin sink (CMRPS). The detailed 3D schematics of the structures are provided in Figure 1. Using the RMRPS as an example, the overall geometrical dimensions and the coordinate system are described. The rectangular manifold has a total length of 6 mm and a width of 0.5 mm. The trapezoidal manifold introduces a 1.6° inclination along the long side relative to the rectangular manifold, as can balance the thermal performance and temperature uniformity of heat sink. Furthermore, the curved rectangular manifold is derived from the trapezoidal version by displacing the midpoints of both long sides by 0.03 mm toward the cold flow end. As shown in Figure 2a, the structures of different manifolds are presented.

The geometric dimensions of the straight rectangular channel and the rhombic pin-fin are illustrated in Figure 2. The rectangular fin has a width of $w_{srf}$ = 0.07 mm and a height of $h_{srf}$ = 0.5 mm, while the total length of the microchannel is 6 mm. To facilitate comparison of the thermal performance among different MCHSs, the channel width in the SRS is set equal to the gap between two adjacent rhombic pin-fins in the RPS. The height of the rhombic pin-fin will be specified separately for the RPS and MRPS in subsequent sections. Detailed geometric parameters of the pin-fins and manifolds are provided in Table 1.

## 3. Numerical Models

Three-dimensional multi-physics field model of COMSOL 6.0 was employed to simulate the flow and heat transfer in MCHSs in a steady state. To simplify the model, the following assumptions are made:

(1) The fluid in MCHS is a stable (Re<960) and incompressible, (2) the physical properties of the fluid and the solid remain constant, (3) no slip at the interface of fluid-solid, (4) the heat loss due to natural convection and radiation is ignored, (5) the energy loss due to viscosity dissipation is also ignored.

### 3.1. Boundary Conditions and Control Equations

Based on the above assumptions, the control equations for the continuity equation, Momentum equation, and the energy equation can be written as follows:

Continuity equation:

(1) ∇·u=0

Momentum equation:

(2) $$(u\cdot \nabla )\rho_{f}u=-\nabla P+\mu \nabla^{2}u$$

Energy equation:

(3) $$\rho_{f}c_{p,f}(u\cdot \nabla T)=k_{f}\nabla^{2}T$$

Heat conduction equation in solid:

(4) $$\nabla \cdot (k_{s}\nabla T)=0$$

**Figure 3:**
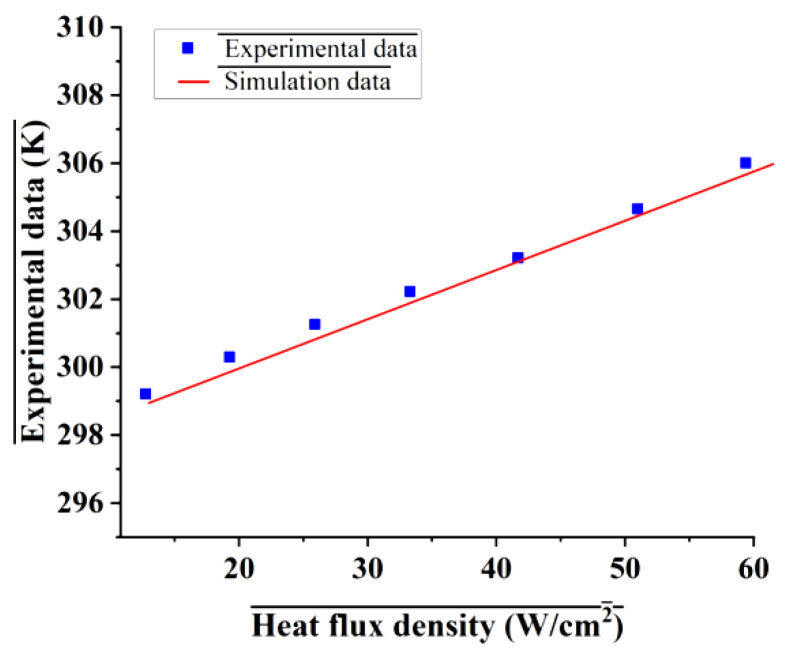
Comparison of experimental results and simulation results of bottom temperature at different heat flux and different flow rate.

**Figure 4:**
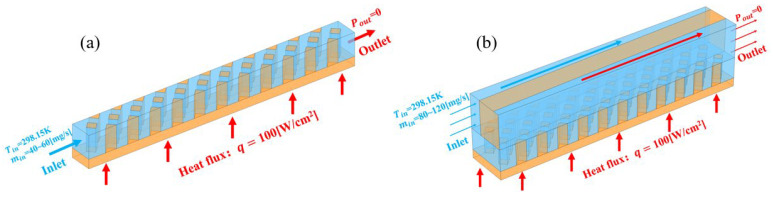
Boundary condition settings, blue and orange represent liquid and solid (**a**) RPS; (**b**) TMRPS.

At the fluid-solid interface, the boundary conditions are:

(5) $$T_{s,\Gamma }\;=\;T_{f,\Gamma }$$

(6) $$k_{s}\frac{\partial T_{s}}{\partial n}{|}_{\Gamma }=\;k_{f}\frac{\partial T_{f}}{\partial n}{|}_{\Gamma }$$

(7) $$u_{s,\Gamma }\;=\;u_{f,\Gamma }\;=\;0$$

where $\rho_{f}$, $c_{f,w}$, $k_{f}$ and $k_{s}$ represent the fluid density, specific heat, thermal conductivity of the fluid and solid, respectively. In this study, deionized water and copper were employed as the coolant and solid material, respectively, their physical properties listed in Table 2 are the same as that in Ref. [29]. The comparison between the experimental results reported in Ref. [29] and the numerical results shown in Figure 3 reveals a maximum relative error of less than 0.461%, confirming the validity and reliability of both the numerical model and the physical property inputs employed. The coolant inlet temperature was set to 298.15 K. For the MCHS, the inlet mass flow rate ranged from 40 to 60 mg/s. Given that the MMC has twice the volume of the MCHS, its inlet mass flow rate was set to 80–120 mg/s to maintain comparable flow conditions. Outflow was set at outlet. A uniform heat flux of 100 W/cm^2^ was applied to the heat source (such as GPU H100 SXM launched by NVIDIA [1]), with heating areas of 0.5 mm × 6 mm for the MCHS and 1 mm × 6 mm for the MMC. All external surfaces, except for the heated substrate, were treated as adiabatic. The boundary condition settings are illustrated schematically in Figure 4, using the RPS and TMRPS configurations as examples.

### 3.2. Data Processing

To characterize the fluid dynamics and evaluate the thermal performance of various heat sinks, some nondimensional parameters should be introduced or defined [30].

(1) Reynolds number:

(8) $$Re=\frac{\rho_{f}u_{m}D_{h}}{\mu }$$

where $\rho_{f}$, $u_{m}$ and μ are the density, average velocity, and dynamic viscosity of deionized water, respectively. $D_{h}$ represents the equivalent diameter of the microchannel, defined as:

(9) $$D_{h}=\frac{2w_{c}h_{p}}{w_{c}+h_{p}}$$

where $w_{c}$ and $h_{p}$ represent the width and height of the microchannel, respectively.

(2) Heat transfer coefficient:

(10) $$h=\frac{Q}{A_{con}\times \Delta T_{ave}}$$

where Q is the heat transferred to the heat sink, defined as $Q=q\cdot A_{sub}$, $A_{sub}$ is the heating area of the substrate, $A_{con}$ is the convective heat transfer area, and $\Delta T_{ave}$ is the difference between the average wall temperature of pin-fin and the average fluid temperature, defined as: $\Delta T_{ave}=T_{W,ave}-T_{f,ave}$.

(3) Nusselt number:

(11) $$Nu=\frac{hD_{h}}{k_{f}}$$

(4) Friction coefficient:

(12) $$f=\frac{2\Delta pD_{h}}{\rho_{f}{u_{m}}^{2}l_{sub}}$$

where Δp, $l_{sub}$ are pressure drop in heat sink and length of substrate.

(5) Performance evaluation criterion:

(13) $$PEC=\frac{Nu}{{\left(f\right)}^{1/3}}$$

(6) Temperature uniformity:

(14) $$\sigma_{T}=\sqrt{\frac{1}{N}\sum_{i=1}^{N}\;(T_{i}-\mu_{T}{)}^{2}}$$

where $T_{i}$ and $\mu_{T}$ represent the local temperature and average temperature on the heat source surface.

### 3.3. Model Validation and Grid Independence Verification

**Figure 5:**
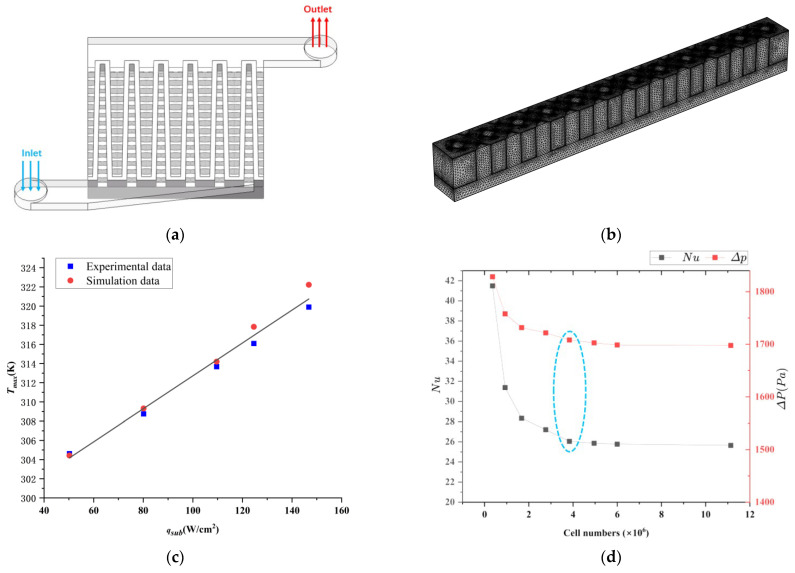
Geometric model for MMC (**a**), mesh generation, the mesh numbers in blue circle are selected for our computation, (**b**), comparison of numerical and experimental results [29] for validation (**c**), verification of grid independence (**d**).

The numerical model was validated against the geometric model and experimental data from Ref. [29]. Figure 5a depicts the geometry of MMC. The numerical results show good agreement with the experimental data, the maximum discrepancy of maximum temperature is 0.725% (Figure 5c), confirming the model’s validity. The increase in error at higher heat fluxes is attributed to the model’s neglect of natural convection and radiation heat transfer.

To verify grid independence, the Nusselt number and pressure drop were computed for a RPS using eight different grid resolutions. The operating conditions were set to an inlet flow rate of 40 mg/s, a rhombic inlet angle of 90°, and a rhombic side length of 0.17 mm. The results, presented in Figure 5d, indicate that for grids with more than 3.83474 × 10^6^, 0.92703 × 10^6^, 1.67384 × 10^6^, 2.76342 × 10^6^, 3.83474 × 10^6^, 4.95068 × 10^6^, 5.99493 × 10^6^, 1.113511 × 10^7^, the changes in the Nusselt number and pressure drop are negligible (below 0.609% and 1.568%, respectively). This demonstrates that the solution is grid-independent, thereby ensuring the accuracy and reliability of the simulations (see that marked by blue).

## 4. Results and Discussions

### 4.1. Comparison of Thermal Performance of Various Micro Heat Sinks

Six micro heat sinks, SRS, RPS, TMSRS, RMRPS, TMRPS and CMRPS, were simulated for flow and heat transfer. Note, the last four sinks have different manifolds. The width and height of microchannel for SRS are $w_{p}$ = 0.18 mm and $h_{p}$ = 0.5 mm, respectively. The inlet angle, side length and height of rhombic pin-fin are set to be $\theta_{p}\;$= 90°, $w_{p}\;$= 0.1736 mm and $h_{p}\;$= 0.5 mm, respectively, for ensuring the same channel width of RPS and SRS. The height ratio between the manifold and pin-fin was set to 1.6 for all four MMCs. The geometric dimensions of the rectangular channel and the rhombic pin-fin were kept consistent with those of the SRS and RPS. Five inlet mass flow rates—40 mg/s, 45 mg/s, 50 mg/s, 55 mg/s, and 60 mg/s—were considered for SRS and RPS in the simulations. Correspondingly, the inlet flow rates for the four MMCs were set to twice those values: 80 mg/s, 90 mg/s, 100 mg/s, 110 mg/s, and 120 mg/s.

**Figure 6:**
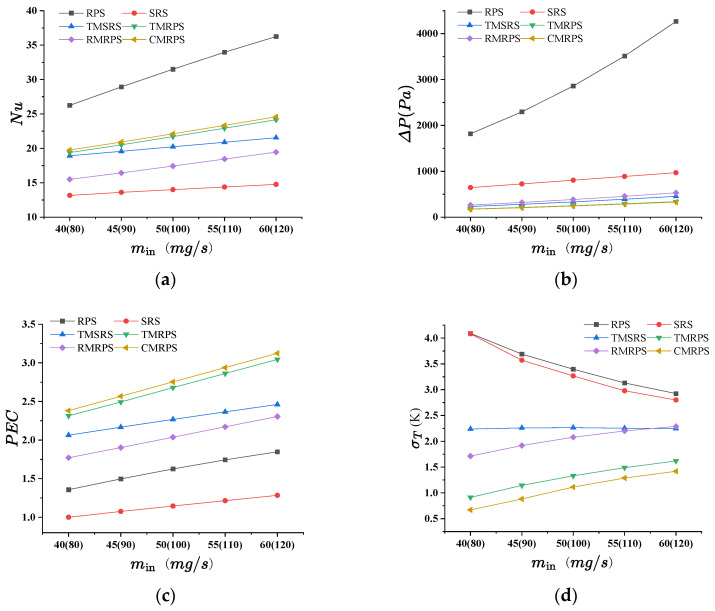
Comparison of heat transfer performance among various types of microchannel heat sinks: (**a**) Nu, (**b**) Δp, (**c**) *PEC*, (**d**) $\sigma_{T}$.

**Figure 7:**
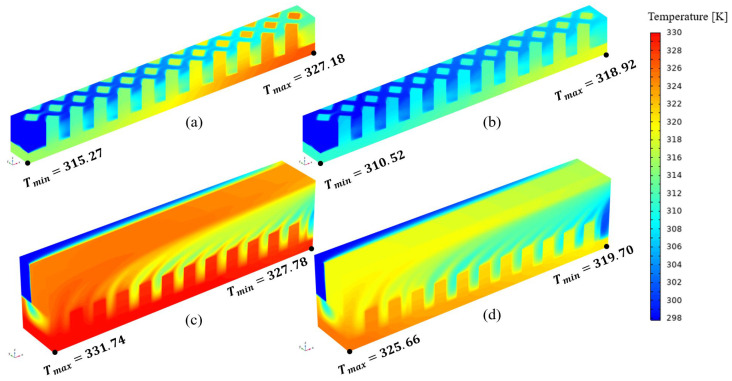
Temperature distributions for four cases. (**a**) RPS (40 mg/s); (**b**) RPS (60 mg/s); (**c**) TMRPS (80 mg/s); (**d**) TMRPS (120 mg/s).

As shown in Figure 6, both the Nusselt number and pressure drop increase with the inlet flow rate. Figure 6a indicates that the Nusselt number is highest for RPS and lowest for SRS. This is attributed to the pin-fin structure, which enhances convective heat transfer and periodically disrupts the thermal boundary layer [14]. A comparison between SRS and TMSRS reveals that the manifold alters the flow pattern and shortens the flow path, leading to a thinner thermal boundary layer and thereby improving heat transfer. Figure 6b shows that the pressure losses in the MMCs are significantly lower than those in the heat sinks without a manifold, which can be ascribed to the shorter flow path in the MMC configurations [21]. Figure 6c illustrates that the performance evaluation criterion (PEC) increases with the flow rate for all six models. The PEC values of the four MMCs are notably higher than those without a manifold. In particular, TMRPS and CMRPS exhibit the highest PEC values, owing to the effective flow distribution enabled by the trapezoidal and curved manifold designs [25]. In contrast, a different trend is observed for temperature uniformity, as shown in Figure 6d. While temperature uniformity improves with increasing flow rate in heat sinks without a manifold, the opposite occurs in those with a manifold. Nevertheless, overall, the MMCs exhibit better temperature uniformity. A comparison of the temperature fields of RPS and TMRPS in Figure 7 shows that the highest temperature in RPS appears near the outlet, whereas in TMRPS it is located near the inlet. This phenomenon can be attributed to the fact that the broader inlet of the trapezoidal manifold is capable of introducing only a limited volume of fluid into the heat sink with rhombic pin-fins. Consequently, this leads to an elevated temperature in proximity to the inlet. Moreover, the volume of fluid entering the heat sink at the upstream decreases with an increase in the inlet flow rate, while the volume entering the heat sink at the downstream conversely increases.

### 4.2. Effect of Pin-Fin on the Thermal Performance for RPS

**Figure 8:**
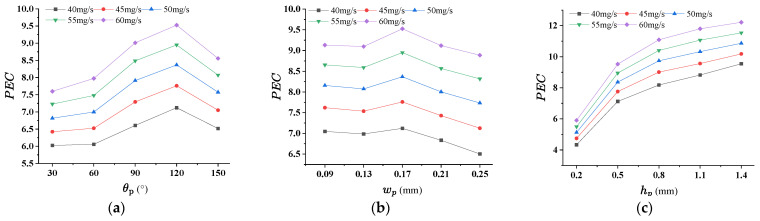
The effect of pin-fin structural parameters on PEC for RPS. (**a**) $\theta_{p}$ ($w_{p}$ = 0.17 mm, $h_{p}\;$= 0.5 mm); (**b**) $w_{p}\;$($\theta_{p}\;$= 120°, $h_{p}\;$= 0.5 mm); (**c**) $h_{p}\;$($\theta_{p}\;$= 120°, $w_{p}\;$= 0.17 mm).

**Figure 9:**
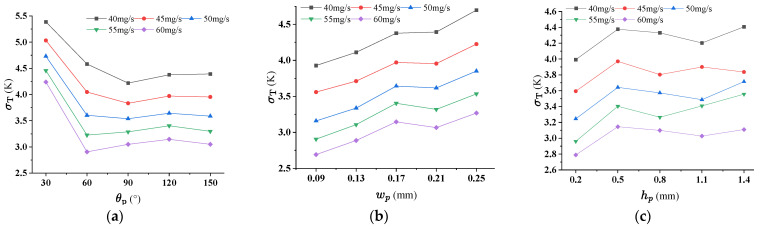
The effect of pin-fin structural parameters on $\sigma_{T}$ for RPS. (**a**) $\theta_{p}\;$($w_{p}\;$= 0.17 mm, $h_{p}\;$= 0.5 mm); (**b**) $w_{p}\;$($\theta_{p}\;$= 120°, $h_{p}\;$= 0.5 mm); (**c**) $h_{p}\;$($\theta_{p}\;$= 120°, $w_{p}\;$= 0.17 mm).

**Figure 10:**
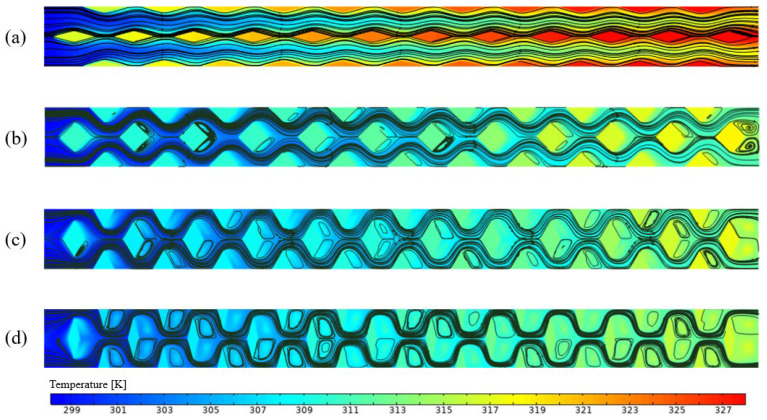
Temperature contours and streamlines at the plan near heated surface in RPS. ($m_{in}$ = 60 mg/s, $w_{p}\;$= 0.17 mm, $h_{p}\;$= 0.5 mm), (**a**) $\theta_{p}\;$= 30°; (**b**) $\theta_{p}\;$= 90°; (**c**) $\theta_{p}\;$= 120°; (**d**) $\theta_{p}\;$= 150°.

Three geometric parameters, inlet angel $\theta_{p}$, side length $w_{p}$ and height $h_{p}$ of rhombic pin-fin, were considered in this section. Numerical simulations for RPS were carried out for calculating the PEC and $\sigma_{T}$ when the inlet flow rate being 40 mg/s, 45 mg/s, 50 mg/s, 55 mg/s and 60 mg/s. Figure 8a–c indicates that the maximum values of PEC occurs at $\theta_{p}\;$= 120°, $w_{p}\;$= 0.17 mm, $h_{p}\;$= 1.4 mm, for all flow rates. Figure 9a–c demonstrates that, lower $\sigma_{T}$ response to >60°, smallest $w_{p}$ and $h_{p}\;$> 0.5 mm. After comprehensive consideration, the preferential results are $\theta_{p}\;$= 120°, $w_{p}\;$= 0.17 mm, $h_{p}\;$= 1.1 mm. The consideration for option $h_{p}\;$= 1.1 mm is that the temperature uniformity is the best. Increasing $\theta_{p}$ leads to a gradual increase in both the Nusselt number and pressure drop. This trend arises because, at a fixed $w_{p}$, an increase in $\theta_{p}$ reduces the streamwise spacing between adjacent rows of staggered pin-fins while increasing the spanwise spacing between columns. As the fluid flows through the inter-fin gaps, coherent vortices form in the wake region between successive rows of rhombic pin-fins (Figure 10). A larger $\theta_{p}$ intensifies these vortices, thereby enhancing momentum and energy transportation—ultimately strengthening convective heat transfer performance.

### 4.3. Effect of Pin-Fin on the Thermal Performance for TMRPS

**Figure 11:**
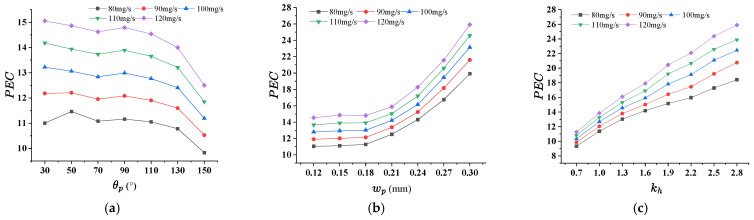
The effect of pin-fin structural parameters on PEC for TMRPS. (**a**) $\theta_{p}$ ($w_{p}\;$= 0.17 mm, $k_{h}\;$= 1.6); (**b**) $w_{p}\;$($\theta_{p}\;$= 90°, $k_{h}\;$= 1.6); (**c**) $k_{h\;}$($\theta_{p}\;$= 90°, $w_{p}\;$= 0.24 mm).

**Figure 12:**
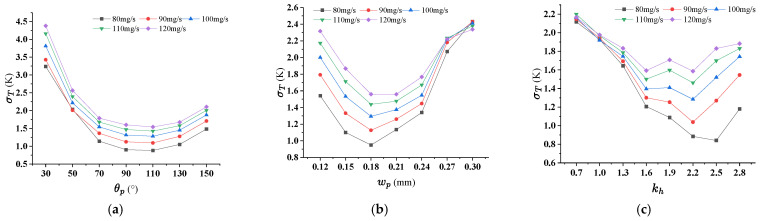
The effect of pin-fin structural parameters on $\sigma_{T}$ for TMRPS. (**a**) $\theta_{p}$ ($w_{p}\;$= 0.17 mm, $k_{h}\;$= 1.6); (**b**) $w_{p}\;$($\theta_{p}\;$= 90°, $k_{h}\;$= 1.6); (**c**) $k_{h\;}$($\theta_{p}\;$= 90°, $w_{p}\;$= 0.24 mm).

**Figure 13:**
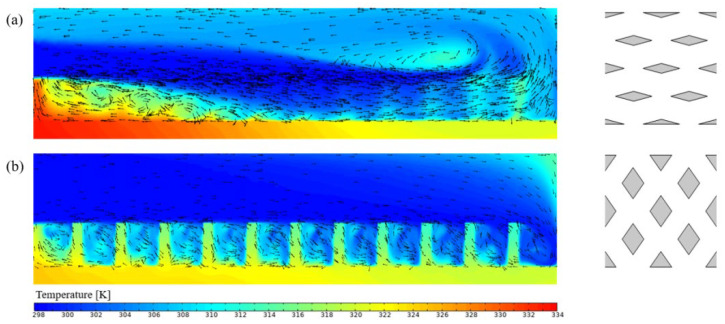
The temperature contour(indicated by colors) and velocity vector(velocity direction indicated by arrows) at the plane of Y = 0.93 mm ($m_{in}$ = 120 mg/s, $w_{p}$ = 0.17 mm, $k_{h}$ = 1.6): (**a**) $\theta_{p}\;$= 30°; (**b**) $\theta_{p}\;$= 110°.

Like that in last subsection, three geometric parameters, inlet angel $\theta_{p}$, side length of rhombic pin-fin $w_{p}$ and ratio of height of manifold to rhombic pin-fin $k_{h}$ of rhombic pin-fin, were considered in this section. Numerical simulations for TMRPS were carried out for calculating the *PEC* and $\sigma_{T}$ when the inlet flow rate being 80 mg/s, 90 mg/s, 100 mg/s, 110 mg/s and 120 mg/s. Figure 11a–c indicates that the maximum values of PEC occurs at smaller $\theta_{p}$, greater $w_{p}$ and $h_{p}$, for all flow rates. Figure 12a–c demonstrates that, lower $\sigma_{T}$ response to $\theta_{p}$ = 90°, smallest $w_{p}=0.18$ and $k_{h}\;$= 2.2 mm. When $\theta_{p}\;$= 30°(Figure 13a), from the rhombic arrangement, it can be seen that the distance between the pin-fins is relatively larger. The fluid in the manifold directly impacts the rear portions of the microchannel, enhancing the heat transfer performance. However, the fluids entrancing the pin-fins array near the inlet is insufficient, and the heat transfer performance is not so good. Hence, the temperature uniformity of the heated surface is worse. When $\theta_{p}$ = 110° (Figure 13b), instead, the channel width decreases, while the space of two rows of pin-fins increases. The fluid entering the front portions of the microchannel increases, and the temperature uniformity can be significantly improved.

**Figure 14:**
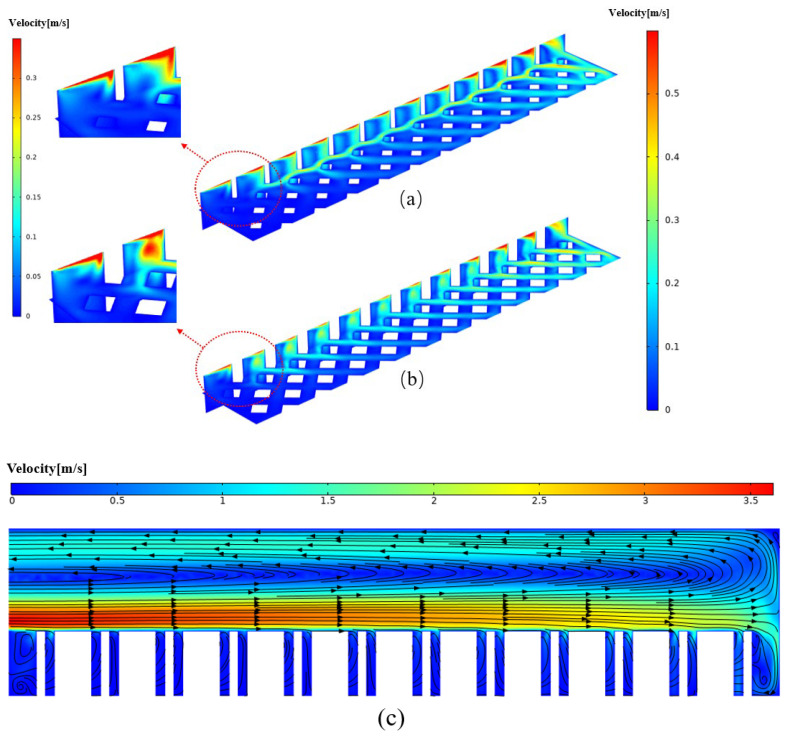
The velocity contours and streamlines at Y = 0.9 mm, Z = 0.45 mm, the colors represent velocity magnitude, and the arrows represent the velocity direction. ($m_{in}$ = 110 mg/s, $\theta_{p}\;$= 90°, $k_{h}\;$= 1.6): (**a**) $w_{p}\;$= 0.12 mm; (**b**) $w_{p}\;$= 0.18 mm; (**c**) $w_{p}\;$= 0.3 mm.

The velocity fields at Y = 0.9 mm and Z = 0.45 mm, shown in Figure 14a, indicate that a smaller $w_{p}$ results in a greater temperature difference between the front and rear portions of the microchannel heat sink. This occurs because fewer fluids enter the front portion, while more enter the rear portion. Conversely, as shown in Figure 14b, a larger $w_{p}$ leads to a greater temperature difference because fewer fluids enter the rear portion, while more enter the front portion. When $w_{p}$ exceeds 0.18 mm, the flow resistance within the pin-fins array increases. Consequently, more fluids flow directly downward through the manifold and enter the rear portion of the microchannel heat sink, which deteriorates temperature uniformity. For example, at $w_{p}\;$= 0.3 mm, the space between two rows of pin-fins is 0.0535 mm, and significant backflow can be observed, as illustrated in Figure 14c.

**Figure 15:**
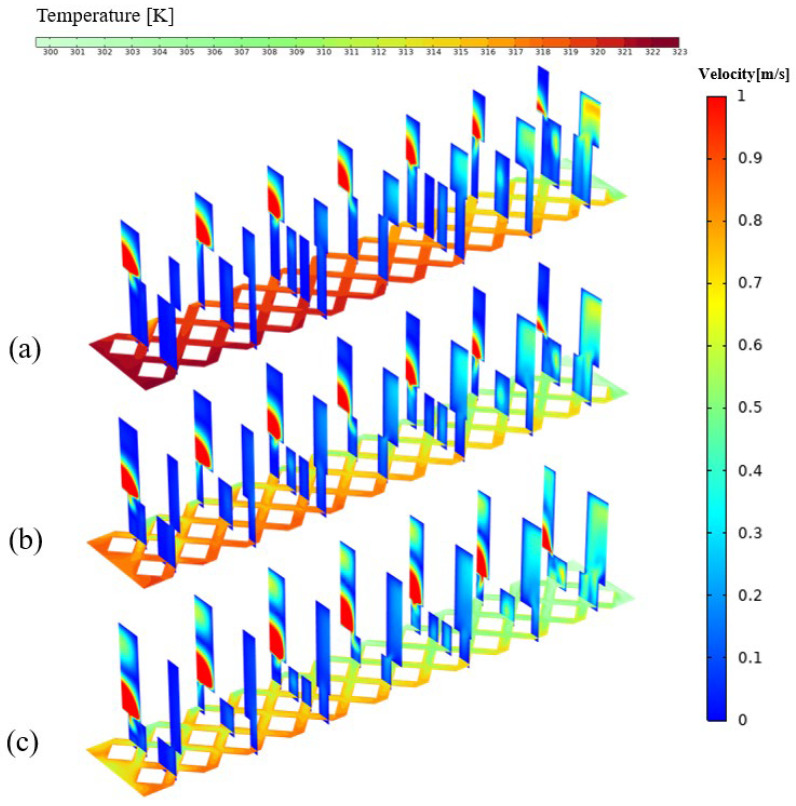
The 7 velocity contours at various planes along the stream and temperature contours of heat sink. (at the plane Z = 0.05 mm) for various height ratio $k_{h}$ ($m_{in}\;$= 120 mg/s, $\theta_{p}\;$= 90°, $w_{p}\;$= 0.24 mm) (**a**) $k_{h}\;$= 0.7; (**b**) $k_{h}\;$= 1.6; (**c**)${\;k}_{h}\;$= 2.8.

As shown in Figure 15, the maximum temperature of the heat sink decreases as the height ratio increases. This occurs because, at a lower pin-fin height, the flow resistance is relatively small, allowing fluid to enter the heat sink more easily, which improves cooling performance and enhances temperature uniformity. However, when the height ratio increases beyond a certain point, backflow occurs in the manifold (Figure 13), leading to an elevated temperature rise at the inlet and a consequent reduction in temperature uniformity. After comprehensive consideration, the preferential results are $\theta_{p}\;$= 90°, $w_{p}\;$= 0.24 mm, $k_{h}\;$= 2.2 mm. The option $w_{p}\;$= 0.24 mm, $k_{h\;}$= 2.2 mm is to compromise some degree of temperature uniformity (before serious deterioration) in pursuit of a higher PEC.

### 4.4. Structural Optimization of TMRPS

The results presented in the previous subsection demonstrate the superior thermal performance and temperature uniformity of TMRPS. To further enhance performance, a multi-objective optimization is performed in this section. The optimization focuses on three design variables ($\theta_{p}$, $w_{p}$, $k_{h}$) with two objectives, maximum PEC, minimum $\sigma_{T}$ under the constrain $T_{max}$ < 343.15 K.

#### 4.4.1. Verification of the Kriging Surrogate Model

**Figure 16:**
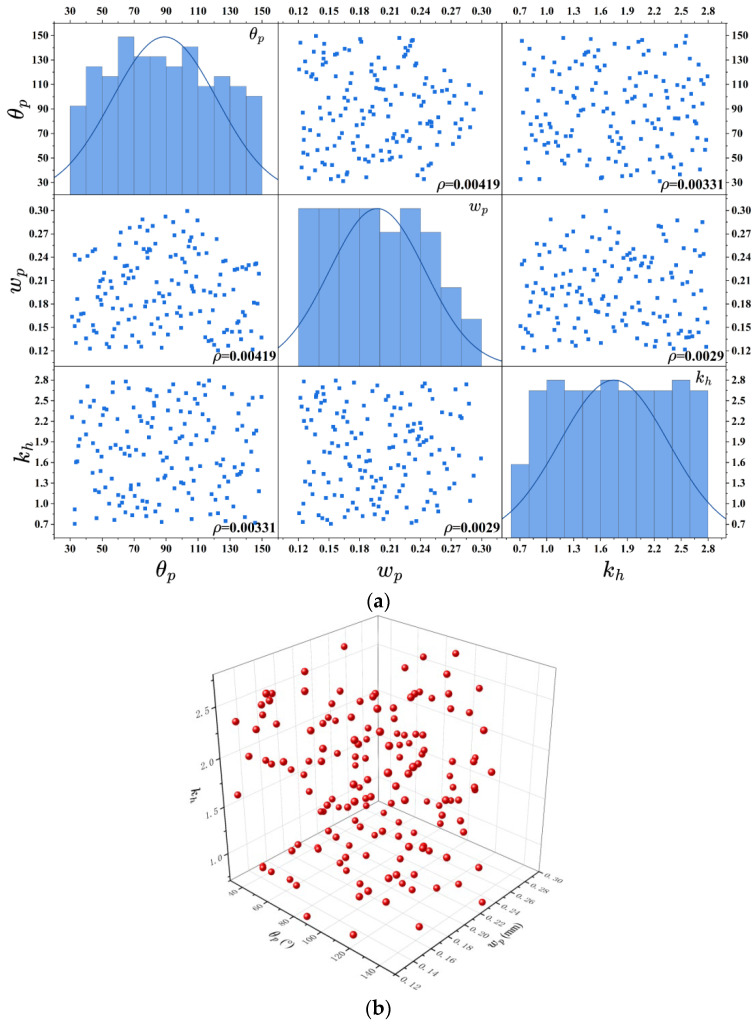
DOE sample points. (**a**) Parameter correlation coefficient matrix diagram; (**b**) spatial distribution of sample points.

The numerical simulation of the TMRPS utilizes a mesh exceeding eight million cells. Employing this high-fidelity model directly for optimization would be computationally prohibitive. Therefore, an accurate surrogate model is adopted to approximate the physical system during the optimization process. The Kriging model is selected for this task due to its high accuracy, strong generalization capability, and inherent robustness. Prior to optimization, a set of design of experiments (DOE) samples was generated to train the Kriging surrogate model. The quality of the DOE is critical and was evaluated based on two key metrics: the correlation coefficients between parameters and the minimum Euclidean distance between sample points. Given the strong nonlinearity of the TMRPS objective function, the Latin hypercube sampling (LHS) method was enhanced with a simulated annealing algorithm. This approach was used to maximize the minimum distance and minimize the correlation coefficients, thereby ensuring uniform distribution and orthogonality of the samples. The sampling ranges for the design variables are:$\;\theta_{p}=\;$30–150°, $w_{p}=\;$0.12–0.3 mm, $k_{h}=\;$0.7–2.8. And the following geometric constraints were applied to ensure model convergence: $w_{p}\cdot \sin\left(\frac{\theta_{p}}{2}\right)\leq 0.235\;mm$; $w_{p}\cdot \cos\left(\frac{\theta_{p}}{2}\right)\leq 0.235\;mm$. Figure 16 presents the correlation coefficient matrix and the spatial distribution of the 150 generated sample points. The results confirm a high-quality DOE, with a maximum absolute correlation coefficient of just 0.004 and a minimum Euclidean distance of 0.07775, indicating excellent uniformity and orthogonality.

With the inlet mass flow rate fixed at 120 mg/s, the surrogate model was trained using data from numerical simulations, including temperatures at each sample point and the peak temperature on the heat source. The model’s accuracy and generalization ability were then validated by comparing its predictions against actual values for 10 randomly selected points. The results, detailed in Table 3, show maximum relative errors of only 4.717%, 0.162%, and 5.381% for the three objective functions. This high level of accuracy demonstrates that the surrogate model is reliable for use in the subsequent optimization algorithm.

#### 4.4.2. Optimization Results of TMRPS

The Kriging surrogate models with three objective functions were substituted into the Non-dominated Sorting Genetic Algorithm-II (NSGA-II). The parametric study in Section 3.3 revealed that the correlation between the PEC and $\sigma_{T}$ in TMRPS is not strictly positive. When the pin-fin parameter is beyond a certain value, temperature uniformity begins to deteriorate. This makes the acquisition of a Pareto-optimal solution set very essential. The optimization was subject to two types of constraints. First, the geometric constraints were maintained from the initial sampling algorithm. Second, a critical temperature constraint was applied, limiting the maximum temperature to 70 °C to reflect the standard operational limit for electronic devices. The detailed parameters for the NSGA-II optimization algorithm are provided in Table 4. A population size of 200 and a maximum iteration number of 8000 yield a stable Pareto solution set, as this configuration balances search capability with computational efficiency. A crossover probability of 0.9 combined with a mutation probability of 0.1 promotes global search and maintains population diversity, which helps prevent convergence to a local optimum. Furthermore, a Pareto selection ratio of 0.8 is applied, ensuring that a larger proportion of non-dominated individuals is retained in each generation. This enhances the richness and continuity of the Pareto frontier solution set.

**Figure 17:**
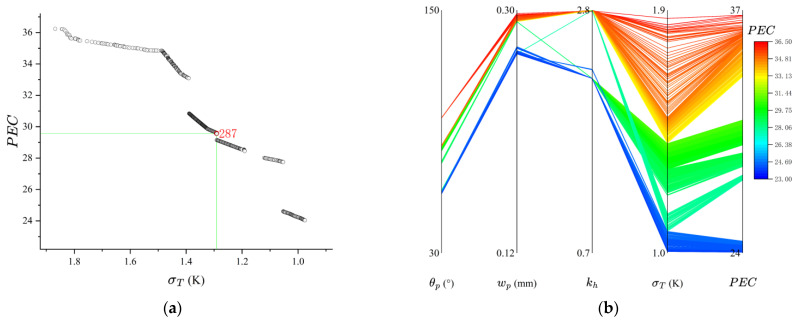
Pareto solution set as a scatter plot (**a**) and parallel coordinate plots (**b**).

To avoid redundant solutions, three decimal places were added for duplicate removal in the Pareto front solution set screening. Figure 17a,b presents the resulting Pareto solution set as a scatter plot, and parallel coordinate plots of the design parameters. The scatter plot in Figure 17a demonstrates that the NSGA-II optimization yielded significant improvements in both the PEC and $\sigma_{T}$. The parallel coordinate plots reveal the optimal parameter ranges: $\theta_{p}$ = 60–100°, combined with larger values for $w_{p}$ and $k_{h}$. and a slightly smaller value for $\theta_{p}$, $w_{p}$ and $k_{h}$ are conducive to better temperature uniformity. Conversely, larger values for $\theta_{p}$, $w_{p}$ and $k_{h}$ lead to a higher PEC. Figure 17b indicates that parameters corresponding to the color spectrum between dark green and orange achieve a balanced compromise between PEC and $\sigma_{T}$.

For validation, Solution 287 from Figure 17a was selected and its parameters were input into the numerical model. The results, summarized in Table 5, confirm the effectiveness of the optimization. At a flow rate of 120 mg/s, the NSGA-II-optimized design increased the PEC by 34.05% and reduced the $\sigma_{T}$ by 18.6% compared to the initial parametric design. Furthermore, when compared to a traditional TMSRS, the optimized design improved PEC by 42.49% and temperature uniformity by 150.75%.

## 5. Conclusions

This study proposes a trapezoidal manifold pin-fin micro-heat sink. The thermal performance of six different micro-heat sink designs was compared across a range of flow rates. We investigated the influence of key geometric parameters—including the rhombic inlet angle, side length, and the manifold-to-pin-fin height ratio—on the heat transfer performance and temperature uniformity of both a standard RPS and the proposed TMRPS. To further enhance the TMRPS’s PEC and temperature uniformity, a multi-objective optimization was conducted. This process employed Latin hypercube sampling, a Kriging surrogate model, and the NSGA-II algorithm to optimize three design variables. The following conclusions can be drawn:

(1) The RPS has better thermal performance compared to the traditional flat SRS. The introduction of the manifold can significantly reduce pressure drop and evenly distribute the flow rate, improving the temperature uniformity of the heat sink. Compared to the rectangular manifold, the trapezoidal manifold distributes the flow rate more evenly, thus being higher PEC and smaller $\sigma_{T}$.
(2) By taking a comprehensive consideration of the maximum PEC and minimum $\sigma_{T}$, the preferential results for RPS and TMRPS are $\theta_{p}\;$= 120°, $w_{p}\;$= 0.17 mm, $h_{p}\;$= 1.1 mm, and $\theta_{p}\;$= 90°, $w_{p}\;$= 0.24 mm, $k_{h}$ = 2.2 mm, respectively.
(3) The generated Pareto frontier solution indicates that TMRPS should adopt a moderate inlet angle, a larger $w_{p}$ and $k_{h}$ to obtain a larger PEC. Slightly reducing $w_{p}$ and $k_{h}$ can sacrifice a certain amount of PEC to achieve better temperature uniformity. Compared with the parameterized preferential solution, the PEC of TMRPS is increased by 34.05%, and the $\sigma_{T}$. is reduced by 18.6%.

## Figures and Tables

**Table 1:** Geometric parameters of manifold, pin-fin and microchannel.

Parameters	Variable	Values
Width of rectangular straight fin	$w_{srf}$	0.07 mm
Height of rectangular straight fin	$h_{srf}$	0.5 mm
Pitch of two column of rhombic pin-fin	$w_{d}$	0.5 mm
Gap of two pin-fin	$w_{d}/2$	0.25 mm
Inlet angle of rhombic pin-fin	$\theta_{p}$	30–150°
Side length of rhombic pin-fin	$w_{p}$	0.09–0.3 mm
Channel width	$w_{c}$	$\left(w_{d}/2-w_{p}*\sin\left(\theta_{p}/2\right))/(cos(\theta_{p}/2)\right)$
Height of pin-fin	$h_{p}$	0.2–1.4 mm (RPS) or $h_{m}/k_{h}$ (TMRPS)
Height ratio of manifold to pin-fin	$k_{h}$	$h_{m}/h_{p}$, 0.7–2.8
Width of manifold	$w_{m}$	0.5 mm
Height of manifold	$h_{m}$	$h_{p}*k_{h}$
Total height	$h_{t}$	$h_{p}+h_{m}=1.3$ mm
Length of substrate	$l_{sub}$	6 mm
Height of substrate	$h_{sub}$	0.2 mm
Width of substrate	$w_{sub}$	$w_{d}$ (MCHS) or $2w_{d}$ (MMC)

**Table 2:** Material physical properties.

	Deionized Water	Copper
$\rho \;[kg/m^{3}]$	998.2	8978
μ [kg/(m·s)]	0.001003	/
$C_{p}\;[J/(kg\cdot K)]$	4182	381
k [W/(m⋅K)]	0.62	387.6

**Table 3:** Accuracy validation of the Kriging surrogate model.

				Predicted Values			Actual Values			Relative Error		
Group	$\theta_{p}\;$[°]	$w_{p}\;$[mm]	$k_{h}$	PEC	$T_{max}\;$[K]	$\sigma_{T}\;$[K]	PEC	$T_{max}\;$[K]	$\sigma_{T}\;$[K]	PEC	$T_{max}\;$[K]	$\sigma_{T}\;$[K]
1	104.804	0.279	2.302	28.160	316.845	2.032	28.463	317.060	2.066	1.063%	0.068%	1.664%
2	142.681	0.146	0.879	8.657	333.340	2.619	8.780	333.481	2.668	1.401%	0.042%	1.826%
3	105.490	0.176	1.761	15.726	324.332	1.426	15.605	324.651	1.491	0.777%	0.098%	4.368%
4	85.807	0.179	1.329	13.233	325.601	1.774	13.232	325.850	1.749	0.010%	0.076%	1.438%
5	76.128	0.229	1.703	18.228	319.875	1.312	17.569	320.395	1.298	3.751%	0.162%	1.079%
6	118.651	0.126	2.180	17.003	328.902	1.618	16.988	328.951	1.647	0.091%	0.015%	1.749%
7	68.571	0.267	1.947	23.686	315.341	1.379	23.964	315.400	1.392	1.160%	0.019%	0.956%
8	77.313	0.229	1.703	18.122	319.911	1.314	17.305	320.344	1.247	4.717%	0.135%	5.381%
9	99.497	0.193	2.007	17.255	322.697	1.202	17.284	322.680	1.264	0.168%	0.006%	4.883%
10	54.368	0.122	2.255	17.846	334.572	3.314	17.913	334.687	3.266	0.375%	0.034%	1.477%

**Table 4:** Parameters of NSGA-II.

Parameters	Value
Population size	200
Maximum number of Generations	8000
Crossover probability	0.9
Mutation probability	0.1
Pareto fraction	0.8

**Table 5:** Comparison of optimization results.

	$m_{in}$ (mg/s)	$\theta_{p}$ (°)	$w_{p}$$/w_{srf}$ (mm)	$k_{h}$	PEC	$\sigma_{T}$
Typical TMSRS	120	/	0.07	1.6	11.801	2.245
Parameterized optimal solution	120	90	0.24	2.2	22.074	1.586
NSGA-II optimal solutions	120	80.416	0.292	2.209	29.591	1.291
Comparison with TMSRS	**—————————————————**				↑150.75%	↓42.49%
Comparison with preferential solution	**—————————————————**				↑34.05%	↓18.6%

## Data Availability

Data are contained within the article.
